# Effects of Shrimp Shell-Derived Chitosan on Growth, Immunity, Intestinal Morphology, and Gene Expression of Nile Tilapia (*Oreochromis niloticus*) Reared in a Biofloc System

**DOI:** 10.3390/md22040150

**Published:** 2024-03-28

**Authors:** Nguyen Vu Linh, Anisa Rilla Lubis, Nguyen Dinh-Hung, Supreya Wannavijit, Napatsorn Montha, Camilla Maria Fontana, Phattawin Lengkidworraphiphat, Orranee Srinual, Won-Kyo Jung, Marina Paolucci, Hien Van Doan

**Affiliations:** 1 Department of Animal and Aquatic Sciences, Faculty of Agriculture, Chiang Mai University, Chiang Mai 50200, Thailand; linhvu.n@cmu.ac.th (N.V.L.); phoooooooooo10@gmail.com (S.W.); napatsorn.m@cmu.ac.th (N.M.); camillamaria.fontana@phd.unipd.it (C.M.F.); orranee.s@cmu.ac.th (O.S.); 2 Functional Feed Innovation Center (FuncFeed), Faculty of Agriculture, Chiang Mai University, Chiang Mai 50200, Thailand; 3 Aquaculture Pathology Laboratory, School of Animal & Comparative Biochemical Sciences, The University of Arizona, Tucson, AZ 85721, USA; 4 Multidisciplinary Research Institute, Chiang Mai University, 239 Huay Keaw Rd., Suthep, Muang, Chiang Mai 50200, Thailand; junjira_l@cmu.ac.th; 5 Marine Integrated Biomedical Technology Center, The National Key Research Institutes in Universities, Pukyong National University, Busan 48513, Republic of Korea; wkjung@pknu.ac.kr; 6 Department of Science and Technologies, University of Sannio, 82100 Benevento, Italy; paolucci@unisannio.it

**Keywords:** by-product, feed additive, growth performance, immune response, mRNA expression

## Abstract

Chitosan (CH) shows great potential as an immunostimulatory feed additive in aquaculture. This study evaluates the effects of varying dietary CH levels on the growth, immunity, intestinal morphology, and antioxidant status of Nile tilapia (*Oreochromis niloticus*) reared in a biofloc system. Tilapia fingerlings (mean weight 13.54 ± 0.05 g) were fed diets supplemented with 0 (CH0), 5 (CH5), 10 (CH10), 20 (CH20), and 40 (CH40) mL·kg^−1^ of CH for 8 weeks. Parameters were assessed after 4 and 8 weeks. Their final weight was not affected by CH supplementation, but CH at 10 mL·kg^−1^ significantly improved weight gain (WG) and specific growth rate (SGR) compared to the control (*p* < 0.05) at 8 weeks. Skin mucus lysozyme and peroxidase activities were lower in the chitosan-treated groups at weeks 4 and 8. Intestinal villi length and width were enhanced by 10 and 20 mL·kg^−1^ CH compared to the control. However, 40 mL·kg^−1^ CH caused detrimental impacts on the villi and muscular layer. CH supplementation, especially 5–10 mL·kg^−1^, increased liver and intestinal expressions of interleukin 1 (*IL-1*), interleukin 8 (*IL-8*), LPS-binding protein (*LBP*), glutathione reductase (*GSR*), glutathione peroxidase (*GPX*), and glutathione S-transferase (*GST-α*) compared to the control group. Overall, dietary CH at 10 mL·kg^−1^ can effectively promote growth, intestinal morphology, innate immunity, and antioxidant capacity in Nile tilapia fingerlings reared in biofloc systems.

## 1. Introduction

The rapid growth of aquaculture has led to an increased demand for improved diets and feed supplements for farmed fish [1]. Feed represents one of the largest costs for aquaculture producers. Determining the specific nutritional requirements, optimal feeding strategies, and nutrient utilization of each fish species is, therefore, critical to enable sustainable and scalable production [2]. Nile tilapia (*Oreochromis niloticus*) has become one of the most widely farmed aquaculture species because of its rapid growth, ability to adapt to different environments, disease resistance, and high protein content in the flesh [3,4]. Recently, tilapia farming practices have shifted from extensive to intensive commercial production systems [5,6]. However, disease outbreaks have emerged as the main obstacle to sustainable intensive tilapia farming globally. Finding ways to prevent diseases will be crucial to address this challenge as tilapia production continues to intensify [6]. Antibiotics have traditionally been used in commercial fish farms to prevent disease transmission. However, concerns over antibiotic usage in aquaculture have led to investigating replacements to reduce reliance on these treatments [7,8]. This has sparked great interest in finding new, innovative feed additives for tilapia, such as probiotics [9,10], prebiotics [11,12,13], and synbiotics [14,15]. These additives have been reported to enhance growth performance, health status, immune function, antioxidant defenses, and immune-related gene expression in tilapia. Such improvements could positively impact overall production. Replacing antibiotics with alternative feed supplements, particularly bioactive compounds derived from seafood products, may provide health benefits for tilapia while addressing issues surrounding antibiotic use in aquaculture.

Shrimp, one of the many varieties of seafood, is a popular and healthful dietary choice globally. Its output reached 8.25 million metric tons in 2015 and reached 9.66 million metric tons in 2019, with an annual growth rate of 2–3% [16], resulting in 6–8 million tons of waste [17,18]. The majority of shrimp waste is discarded publicly in landfills [19], burned, or dumped into the oceans [20,21]. Only a small portion is utilized as food and feed for animals and aquaculture [22,23]. The shrimp disposal sites could be major sources of offensive odors, as well as dust, gases, and fumes [18,24]. The rapid breakdown of shrimp waste can result in the appearance and spread of infections by flies, mosquitoes, and rats, endangering human health [25,26]. Nonetheless, this waste stream also includes beneficial natural substances, chief among them being chitin, which is an essential component in the production of chitosan [27,28,29]. Chitosan (CH) has been shown to have anti-cancer [30,31], anti-inflammatory [32,33], and neuroprotective [34] activities, in addition to having antioxidant, anti-diabetic, anti-hypertensive, and wound-healing [35] properties. Additionally, chitosan has antibacterial properties against the majority of bacteria, molds, and yeasts [36]. Furthermore, chitosan is a nontoxic, biodegradable, and biocompatible biopolymer. These characteristics make chitosan and its derivatives suitable for usage in a wide range of sectors, including the food, pharmaceutical, and agricultural industries [37,38,39].

Biofloc technology (BFT) has emerged as a sustainable aquaculture practice that enables fishponds to self-nitrify without water exchange [40,41]. In BFT systems, flocs formed from organic particulate matter and diverse microorganisms serve as an in situ food source. Fish can directly consume these protein-rich flocculants, reducing the need for fishmeal and soybean meal in feeds [42,43,44,45]. By substituting commercial diets with biofloc, the risks of mycotoxin and antinutrient exposure are also decreased, lowering feed costs [46,47]. Tilapia is especially well-suited for biofloc farming, as the species can effectively utilize biofloc for nutrition [48,49]. Given the benefits of BFT for tilapia production, this study aimed to evaluate how chitosan feed supplementation influences the growth, immune function, intestinal histology, and expression of key immune-antioxidant genes in Nile tilapia reared in a biofloc system. The overarching goal was to assess the potential of CH as a feed additive for enhancing tilapia health and productivity under sustainable BFT conditions.

## 2. Results

### 2.1. Growth Performance

The growth performance of Nile tilapia fingerlings fed the chitosan supplemented diets is shown in Table 1. After 4 weeks, FW was significantly higher (*p* < 0.05) in the CH10 group compared to the control group, while no significant differences were detected among groups at 8 weeks (*p* > 0.05). Weight gain did not differ significantly between the control and treatments at 4 weeks (*p* > 0.05). However, at 8 weeks, fish fed the CH10 diet showed significantly increased weight gain compared to the control group (*p* < 0.05) (Table 1). The CH10 group also exhibited the highest SGR at both 4 weeks (2.83 ± 0.07) and 8 weeks (2.29 ± 0.03) (Table 1). No significant differences in FCR were observed between groups at any time (Table 1). The survival rate exceeded 95% in all treatments after the 8-week feeding trial. 

### 2.2. Immunological Response

Lysozyme and peroxidase activities of skin mucus in Nile tilapia after 4 and 8 weeks of feeding are shown in Table 2. At both time points, skin mucus lysozyme activity (SMLA) and skin mucus peroxidase activity (SMPA) were significantly higher in the control group compared to all dietary CH treatments (*p* < 0.05). No significant differences were detected between the various CH-supplemented diets for either enzyme activity (*p* > 0.05).

Serum lysozyme and peroxidase activities are illustrated in Table 3. Serum lysozyme activity (SL) was higher in the control group at 4 weeks. Serum peroxidase activity (SP) was significantly higher in CH 10 at week 4 (*p* < 0.05). However, this difference was not significant at 8 weeks. In contrast, the CH20 and CH40 groups exhibited notably reduced SP at both sampling times.

### 2.3. Histological Analysis

**Figure 1:**
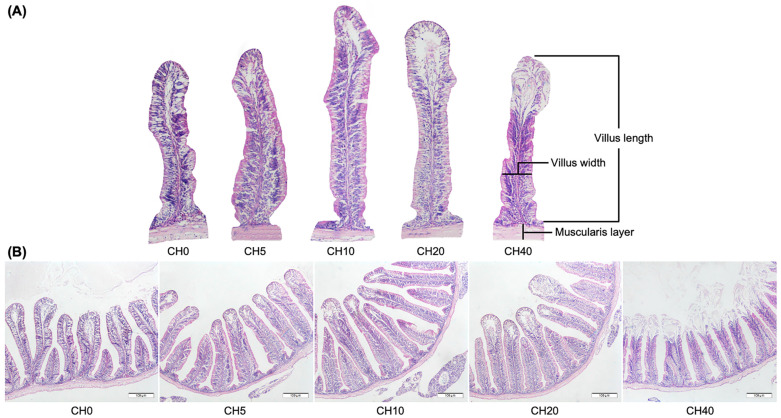
Intestinal morphology in Nile tilapia (*Oreochromis niloticus*) fingerlings fed diets with increasing chitosan (CH) levels after 8 weeks compared to the non-supplemented control diet. (**A**) A comparison of the length and width of the villus and thickness of the muscularis layer. (**B**) The cross-section through the microanatomy of the anterior intestine. Diets: CH0, 0 mL·kg^−1^ COS (control); CH5, 5 mL·kg^−1^; CH10, 10 mL·kg^−1^; CH20, 20 mL·kg^−1^; CH40, 40 mL·kg^−1^ of chitosan. The tissue was stained with hematoxylin and eosin (H&E). The bars in the pictures are 100 µm.

**Figure 2:**
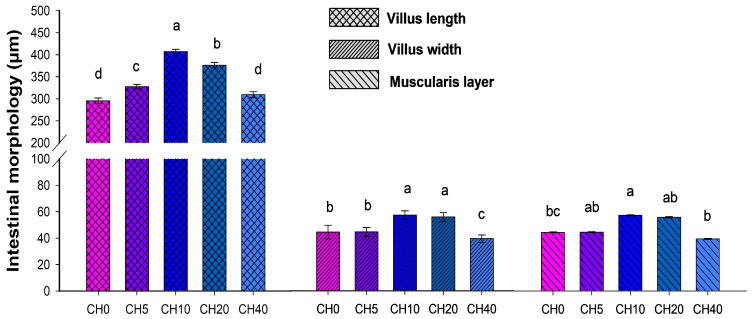
The measurements of the intestinal morphology of Nile tilapia (*Oreochromis niloticus*) fingerlings fed diets with increasing chitosan (CH) levels. Diets: CH0, 0 mL·kg^−1^ COS (control); CH5, 5 mL·kg^−1^; CH10, 10 mL·kg^−1^; CH20, 20 mL·kg^−1^; CH40, 40 mL·kg^−1^ of chitosan. Five sections were randomly selected for measurement for each fish, with three fish per treatment. Values are means ± SEM (*n* = 15, microfields). Values in the same row with different superscripts indicate a significant difference between the CH-containing groups (*p* < 0.05).

Intestinal morphology and related parameters of Nile tilapia fingerlings are presented in Figure 1 and Figure 2. Villus length and width were significantly increased in the CH10 treatment group compared to the control group and the other treatment groups (*p* < 0.05). Fish fed the CH20 diet also exhibited greater villus length and width compared to the control. Additionally, the muscularis layer was the thickest in the CH10 group among all diets (*p* < 0.05). In contrast, the CH40 diet resulted in noticeable morphological alterations, including decreased villus length, villus width, and reduced muscularis thickness (*p* < 0.05). 

### 2.4. Immune and Antioxidant-Related Gene Expressions

**Figure 3:**
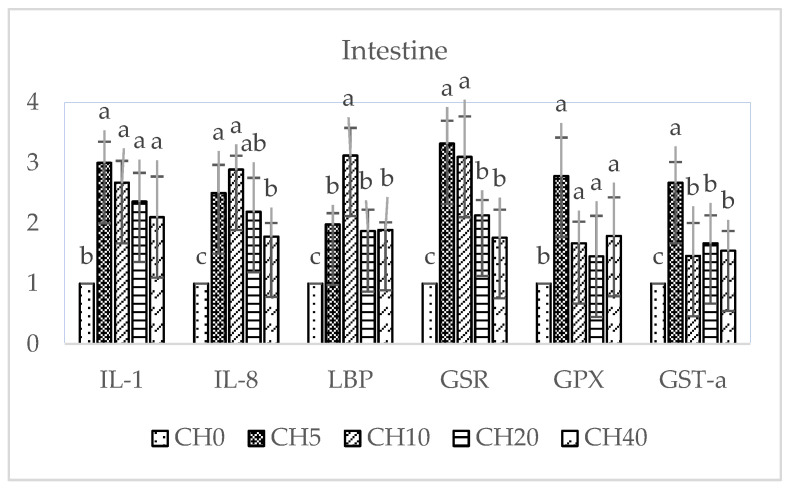
Expression transcript levels of interleukin-1 (*IL-1*), interleukin-8 (*IL-8*), lipopolysaccharide-binding protein (*LBP*), glutathione reductase (*GSR*), glutathione peroxidase (*GPX*), and glutathione S-transferase-α (*GST-α*) in the liver of Nile tilapia (*Oreochromis niloticus*) fingerlings fed diets with increasing chitosan (CH) levels for 4 and 8 weeks (*n* = 6). Data is shown as mean ± SEM. Different letters (a–c) indicate significant differences between dietary groups by one-way ANOVA and Duncan’s test (*p* < 0.05). Diets: CH0, 0 mL·kg^−1^ COS (control); CH5, 5 mL·kg^−1^; CH10, 10 mL·kg^−1^; CH20, 20 mL·kg^−1^; CH40, 40 mL·kg^−1^ of chitosan.

**Figure 4:**
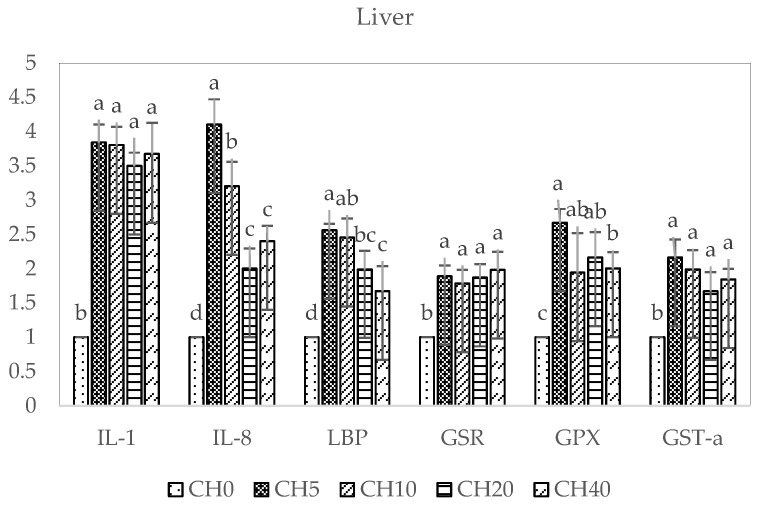
Expression transcript levels of interleukin-1 (*IL-1*), interleukin-8 (*IL-8*), lipopolysaccharide-binding protein (*LBP*), glutathione reductase (*GSR*), glutathione peroxidase (*GPX*), and glutathione S-transferase-α (*GST-α*) in intestine of Nile tilapia (*Oreochromis niloticus*) fingerlings fed diets with increasing chitosan (CH) levels for 4 and 8 weeks (*n* = 6). Data is shown as mean ± SEM. Different letters (a–c) indicate significant differences between dietary groups by one-way ANOVA and Duncan’s test (*p* < 0.05). Diets: CH0, 0 mL·kg^−1^ COS (control); CH5, 5 mL·kg^−1^; CH10, 10 mL·kg^−1^; CH20, 20 mL·kg^−1^; CH40, 40 mL·kg^−1^ of chitosan.

The effects of dietary CH-supplemented on the expression of immune-related (*IL-1*, *IL-8*, *LBP*) and antioxidant-related (*GSR*, *GPX*, *GST-α*) genes in the liver and intestine of tilapia are shown in Figure 3 and Figure 4. In both tissues, all supplemented diets appeared to upregulate these genes compared to the control. In the liver, the CH5 diet induced the greatest increase in most of the genes, with IL-8 expression being significantly higher than the control and the other diets (*p* < 0.05). For *IL-1*, *GSR*, and *GST-α*, there were no significant differences among supplemented groups (*p* > 0.05). *GPX* expression was significantly higher in the CH5 compared to the CH40 group (*p* < 0.05). Additionally, the CH5 and CH10 diet groups elicited increased *LBP* expression compared to the control and CH20 and CH40 groups (*p* < 0.05).

In the intestine, expression of *IL-1* and *GPX* was significantly higher in all dietary CH-treated groups compared to the control group (*p* < 0.05), with no statistically significant differences among supplemented diets (*p* > 0.05). Interestingly, the highest *LBP* expression occurred in the CH10 group, which was significantly different from the control and other treated groups (*p* < 0.05). *GSR* expression was significantly higher in the CH5 and CH10 groups compared to the control and CH20 and CH40 groups (*p* < 0.05). Additionally, CH5 and CH10 diets elicited clear increases in *IL-8* and CH-5 increased *GST-α* expression compared to the control and the other CH-supplemented groups.

## 3. Discussion

The current study demonstrated the beneficial effects of dietary-supplemented CH on the growth of Nile tilapia (*Oreochromis niloticus*) fingerlings cultured in a biofloc system. Influences of dietary chitosan on growth have been evaluated in various aquatic species with variable results [10,50,51,52]. Specifically in Nile tilapia, previous findings on chitosan’s effects as a feed additive have been heterogeneous. Shiau and Yu [53] found that 2–10% of dietary chitosan inhibited tilapia growth, while Romana-Eguia et al. [54] showed no impact on growth. However, other studies [55,56] reported improved growth and meat quality with chitosan supplementation in Nile tilapia. Interestingly, this study indicated that only the 10 mL·kg^−1^ CH diet significantly increased FW, WG, and SGR of Nile tilapia fingerlings, suggesting that the benefits are dose-dependent, with excessive amounts conferring no added growth effects. Indeed, multiple studies have evidenced the detrimental impacts of immunostimulant over-supplementation on aquaculture species, including immune exhaustion and slowed growth [57,58]. Shiau and Yu [53] reported decreased weight gain in Nile tilapia with chitosan, potentially due to reduced nutrient digestibility and absorption. Chitosan particle size may also influence Nile tilapia growth [59]. Several lines of evidence suggest that chito-oligosaccharides can improve growth performance in tilapia through various interrelated mechanisms. As prebiotics, CH can modify intestinal microbial communities in a beneficial manner, supporting gut health and likely enhancing nutrient digestion and absorption [60]. The immunostimulatory effects of chito-oligosaccharides are also thought to play a key role by reducing the susceptibility to infectious diseases, allowing tilapia to allocate more energy towards growth rather than mounting inflammatory responses [61]. Chito-oligosaccharides have additionally been shown to increase the activities of digestive enzymes like protease, lipase, and amylase in tilapia, which could lead to greater utilization of feed for growth [62]. Finally, mitigation of oxidative stress by the antioxidant properties of CH enables available energy to be used for anabolism rather than neutralizing reactive oxygen species, supporting growth [63,64]. The growth-promoting effects of immunostimulants like CH are influenced by numerous factors, including dosage, molecular weight, feeding duration, temperature, administration route, and species difference [65]. Our results demonstrated that the benefits of CH on growth were dose-dependent in Nile tilapia, with 10 mL·kg^−1^ being the optimal supplementation level for improving performance. While the precise modes of action have yet to be elucidated, this study provides valuable insights into appropriate CH dosing strategies for maximizing growth in tilapia aquaculture. Further research is still needed to fully understand the biological pathways and key factors mediating the effects of CH on increasing fish growth rate.

Innate immunity serves as the first line of defense against pathogens in fish [66]. The body’s surface mucosa provides a physical and immunological barrier, playing crucial roles in protection, sensory function, and ion regulation [67,68]. Mucosal responses are key in early infection control, as many pathogens initially adhere to mucosal surfaces during invasion [69]. Lysozyme, found in mucus, fluids, and tissues, is an important component of teleost innate immunity due to its bactericidal and opsonizing effects [70,71]. Serum lysozyme can indicate the innate status of the host by initiating the complement cascade [69,70]. CH’s free radical scavenging amino groups can boost unstable lysozyme [72], and oral chitosan has been shown to increase lysozyme activity in various fish species [73,74]. Peroxidase, another key innate immune enzyme, helps to maintain redox homeostasis in immune cells and acts as a microbicidal agent by destroying H_2_O_2_ [75,76]. In this study, our results demonstrated that dietary CH supplementation at 5–40 mL·kg^−1^ significantly decreased peroxidase and lysozyme activities in the skin mucus after 4 weeks, declining further by 8 weeks. Interestingly, 10 mL·kg^−1^ CH increased blood serum peroxidase activity at 4 and 8 weeks of the feeding trial. These results align with Yu et al. [58], who showed 10 g/kg CH reduced lysozyme in golden pompano (*Trachinotus ovatus*). The contrasting blood and mucus enzyme responses reveal the complex immunomodulatory effects of CH in fish. Achieving optimal benefits likely requires careful dosage optimization. Our findings highlight the need for further research into appropriate CH supplementation strategies to support mucosal and systemic innate defenses in aquaculture species.

Oligosaccharide supplementation in fish diets promotes feed conversion and enhances intestinal microanatomy, improves mucosal epithelium health, and defends against opportunistic bacterial infections [77]. This optimization of intestinal morphology may increase the absorption area of the intestine, facilitating efficient nutrient absorption [78]. In the present study, it was observed that the treatment with CH resulted in an increase in villus height and width, along with an increase in the thickness of the muscularis layer, which was particularly pronounced in the CH10 and CH20 groups. Since the proportion of villi is related to the ability to absorb nutrients through the available surface area, the surface increase could potentially improve nutrient utilization and storage [79]. Our results are consistent with previous studies, such as those showing an increase in villus length following supplementation of hybrid grouper diets with CH oligosaccharides [80] or after supplementation of hybrid catfish diets with mannan oligosaccharides [81]. It has also been reported that various other oligosaccharides can significantly increase villus length in numerous fish species, as shown by 1% galacto-oligosaccharide in the diet for red drum [82] or 2% fructo-oligosaccharide in the diet for bluntnose seabream [83]. However, it is worth noting that the effects of oligosaccharides on the villi structure may vary depending on the fish species, oligosaccharide type and concentration, and the fish species’ own microbiota. In this study, the CH40-containing treatments resulted in some undesirable changes in the intestinal morphology of the fish, including degeneration of the villi and morphological disorders. This outcome may be attributed to an imbalance of amino acids in the diet, possibly triggered by an incorrect ratio when replacing fish meal with CH, which is consistent with previous studies [80,84]. Since dietary amino acids are primarily used to meet growth requirements and build fish tissue, an imbalance of these amino acids may lead to dysplasia in fish intestinal morphology [85,86]. Overall, our results indicate that CH supplementation at 10–20 mL·kg^−1^ may beneficially enhance the intestinal morphology and the absorptive capacity in tilapia, yet higher doses could negatively impact the intestine structure.

Pro-inflammatory cytokine *IL-1* is essential for innate immunity, stimulating lymphocytes, phagocytes, and infection resistance in fish [87]. *IL-8*, released during inflammation, activates inflammatory cells as a neutrophil chemoattractant and mediator [88]. *IL-1* and *IL-8* coordinate innate inflammatory defenses and pathogen clearance by phagocytes [89,90]. The acute-phase protein lipopolysaccharide-binding protein (*LBP*) also has key innate immune functions, binding lipopolysaccharides and eliciting responses to Gram-negative bacteria [91]. In our study, all CH doses markedly increased hepatic and intestinal expression of the immune genes *IL-1*, *IL-8*, and *LBP* compared to the control, indicating activation of innate immune responses in tilapia. These results align with previous observations reporting *IL-1* and *IL-8* upregulation following immunostimulant feeding in tilapia [92]. However, in golden pompano, CH reduced *IL-8* expression [57], highlighting species-specific differences. Antioxidant supplements can improve fish health by reducing oxidative stress. Glutathione peroxidase (*GPX*) and glutathione reductase (*GSR*) remove hydrogen peroxide using glutathione [13,93]. Glutathione S-transferase (*GST*) detoxifies electrophiles, enhancing their elimination [94]. In this study, we found dietary CH-supplemented significantly increased antioxidant gene (*GSR*, *GPX*, *GST-α*) expression in tilapia liver and intestine, similar to previous tilapia studies [13] and golden pompano [57]. This suggests that CH may mitigate oxidative damage. Overall, our gene expression analyses indicate that CH can stimulate innate immune and antioxidant responses in tilapia.

The growth benefits of CH in this study may have been enhanced by using a biofloc production system [95], which consists of suspended microbial biomass that acts as a natural food source [46,96,97,98]. This seems to stem from the fact that the prebiotic effects of CH selectively enriched beneficial biofloc species, maximizing natural productivity. Their immunostimulatory properties likely complemented immune activation by biofloc microbes. The combination of bioavailable nutrients from biofloc consumption and improved digestibility and gut health from CH may have synergistically augmented tilapia growth.

The limitations of this study include the absence of a priori power analysis and the use of non-standard reporting of chitosan concentration in volume units (mL/kg diet) instead of mass units (g/kg diet). The lack of a priori power analysis may serve to hide the true effects of chitosan supplementation, potentially leading to underpowered circumstances that are unable to identify statistically significant results. To address these issues, we propose the use of power analysis in the study design phase as a mean to precisely ascertain the necessary sample sizes. Furthermore, standardizing the reporting of chitosan concentrations in mass units will greatly enhance the reproducibility of research findings and streamline the process of comparing them across different investigations. 

## 4. Materials and Methods

### 4.1. Nile Tilapia Husbandry

Healthy Nile tilapia fingerlings were acquired from a tilapia farm in Chiang Mai Province, Thailand. The fish were first acclimated for two weeks under standard aquaculture conditions and fed on commercial diets twice daily. The tilapia was then moved into fifteen 150 L fiberglass tanks for the feeding trials. Water quality parameters, including temperature (°C), pH, dissolved oxygen (mg·L^−1^), and ammonium, were maintained within optimal ranges for Nile tilapia [99] throughout the experiment as follows: T° = 28.5 ± 0.07; pH = 7.81 ± 0.03; dissolved oxygen = 5.76 ± 0.02 mg·L^−1^, and ammonium = 0.12 ± 0.002 mg·L^−1^. 

### 4.2. Diet Preparation and Experimental Design

#### 4.2.1. Preparation of Chitosan (CH)

The CH supplement used in this study was obtained from Olizac Technologies Co., Ltd., Khlong Nueng, Khlong Luang District, Pathum Thani, Thailand. It was extracted from shrimp shell via enzymatic hydrolysis as described previously [100]. Briefly, shrimp shells underwent deproteinization, demineralization, and depigmentation before being deacetylated with 50% sodium hydroxide to achieve over 90% degree of deacetylation. The CH was then precipitated and lyophilized. A mixture of chitinase and chitosanase enzymes was applied to produce CH with a molecular weight of approximately 10 kDa, as determined by gel permeation chromatography.

#### 4.2.2. Experimental Design

Five experimental diets containing different levels of CH were prepared. The diet formulations are shown in Table 4 The dry ingredients were thoroughly mixed and then pelletized with the addition of oil and water into 2 mm pellets. Feeds were stored at 4 °C until use. After a two-week acclimation, 300 healthy tilapia fingerlings (13.54 ± 0.05 g) were randomly distributed into the following treatment groups (*n* = 20 fish per tank, 3 replicate tanks per treatment): CH0, 0 mL·kg^−1^ CH as control; CH5, 5 mL·kg^−1^; CH10, 10 mL·kg^−1^; CH20, 20 mL·kg^−1^; and CH40, 40 mL·kg^−1^. Fish were fed the experimental diets twice daily for 8 weeks while water quality parameters were monitored daily as described previously [99].

### 4.3. Biofloc Water Preparation

Biofloc was established in the experimental tanks 3 weeks prior to starting the feeding trial. Coarse salt (400 g), molasses (5 g), dolomite (5 g), and control feed (2 g) were added to each tank to initiate floc formation. The carbon-to-nitrogen (C:N) ratio was maintained at 15:1 by supplementing with molasses (40% carbon) 2 h after each feeding [40]. The C/N ratio was monitored by measuring residual nitrogen levels in the tanks and determining the carbon and nitrogen content of the feed.

### 4.4. Growth Performance

After 4 and 8 weeks of feeding the experimental diets, all fish were weighed to assess growth performance. Parameters were calculated as follows:

Weight gain (WG, g) = final weight (FW) − initial weight (IW);

Specific growth rate (SGR, %) = 100 × (ln FW − ln IW)/number of experimental days;

Feed conversion ratio (FCR) = amount of feed given (dry weight)/WG (wet weight);

Survival rate (SR, %) = (final number of fish/initial number of fish) × 100.

### 4.5. Immunological Analysis

#### 4.5.1. Sample Collection

Skin mucus and serum samples were collected to analyze immunological parameters. For skin mucus, 3 fish were randomly selected from each tank and anesthetized with clove oil (200 ppm) to minimize stress and discomfort. Following anesthesia, the fish were humanely euthanized in accordance with ethical guidelines for the collection of skin mucus and serum samples. Individuals were placed in plastic bags containing 10 mL of 50 mM NaCl. The fish were gently rubbed for 1 min to collect skin mucus. The mucus–salt mixture was centrifuged at 1500× *g* for 10 min at 4 °C. The supernatant was stored at −80 °C until analysis.

Blood samples were collected as previously described [99]. Briefly, 1 mL of blood was drawn from the caudal vein of each fish using a 1 mL syringe and immediately transferred into new sterilized tubes (without anticoagulants). Blood samples were kept at room temperature for 1 h and then incubated for 4 h at 4 °C. Serum samples were collected after centrifugation (15 min, 4 °C at 10,000× *g*) and stored at −80 °C until analysis. 

#### 4.5.2. Immunological Parameter Analysis 

Lysozyme and peroxidase activities in undiluted serum and skin mucus samples were performed according to the previously described method [101]. Briefly, 25 μL of serum or 100 μL of skin mucus from each fish was added in triplicate to 96-well plates, followed by 175 μL of a 0.3 mg.mL^−1^ Micrococcus lysodeikticus suspension (in 0.1 M citrate phosphate buffer, pH 5.8). Plates were rapidly mixed, and the decrease in turbidity was measured every 30 s for 10 min at 540 nm using a microplate reader (Synergy H1, BioTek, Santa Clara, CA 95051, USA). A standard curve was generated using known concentrations of hen egg white lysozyme (0–20 μg.mL^−1^, Sigma-Aldrich Inc., St. Louis, MO 68178, USA).

### 4.6. Histopathology Analysis

To examine intestinal morphology, the anterior intestine from 3 fish per treatment was sampled at the end of the trial. Tissues were fixed in 10% neutral buffered formalin for 24 h, then transferred to 70% ethanol. Samples were processed using an automated tissue processor, involving dehydration in graded ethanol, clearing with xylene, and embedding in paraffin wax. The tissues were sectioned at 4–5 μm thickness using a microtome (Leica Biosystems, Deer Park, IL 60010, USA) and stained with hematoxylin and eosin (H and E). Slides were viewed and photographed using a light microscope (BX51 Olympus, Tokyo, Japan). Morphometric analysis was performed by measuring villus length, villus width, and muscularis thickness on 5 randomly selected microfields per fish.

### 4.7. Quantitative Real-Time PCR (qPCR) 

#### 4.7.1. Tissue Sampling, Total RNA Isolation, and cDNA Synthesis

Expressions of immune-related (*IL-1*, *IL-8*, and *LBP*) and antioxidant-related (*GST-α*, *GPX*, and *GSR*) genes were analyzed in the liver and intestine after 8 weeks. Examined organs (20–40 mg) were collected from two fish in each tank (*n* = 6) and stored in sterilized tubes supplemented with 500 µL Trizol (Invitrogen, Waltham, MA, USA) at −80 °C for further analysis. Total RNA was isolated using the PureLink^TM^ RNA Mini Kit (Invitrogen, Thermo Fisher Scientific, Waltham, MA, USA) following the manufacturer’s protocol. RNA quantity and quality were assessed by spectrophotometry (NanoDrop^TM^ 2000, Thermo Scientific, Waltham, MA, USA). One μg of total RNA was used for cDNA synthesis with the iScript^TM^ cDNA kit (BIO-RAD, Hercules, CA, USA).

#### 4.7.2. Quantitative Real-Time PCR

Gene expression was quantified by qPCR using the primer sequences listed in Table 5. Reactions contained 1 μL cDNA (100 ng), 0.4 μL each primer (10 μM), 10 μL 2× SYBR Green Mastermix (BIO-RAD, USA) and nuclease-free water to 20 μL total volume. qPCR was performed on a CFX ConnectTM system (BIO-RAD, USA) as described previously [13]. Relative mRNA levels were calculated using the 2^−ΔΔCt^ method [102] with *18S rRNA* as the internal reference gene.

### 4.8. Statistical Analyses

The Shapiro–Wilk test was used to assess the normality of the data. One-way analysis of variance (ANOVA) was performed to determine statistically significant differences among the dietary treatment groups. The distribution of the sample variables was considered normal (*p* > 0.05) and was evaluated using a one-way ANOVA. Statistical significance among groups (*p* < 0.05) was compared using post hoc LSD analysis and non-normal distribution (*p* < 0.05). All data were analyzed using Statistix (Analytical Software, v10.0 Tallahassee, FL 32312, USA) statistical software.

## 5. Conclusions

In summary, this study demonstrates that dietary supplementation with 10 mL·kg^−1^ CH can effectively improve the growth, health, and productivity of Nile tilapia fingerlings reared in biofloc systems. CH also stimulated innate immunity, as shown by increased serum peroxidase activity at 4 weeks. Most notably, CH feeding markedly upregulated the expression of immune and antioxidant genes in the liver and intestine. This indicates that CH can beneficially modulate the immune status and oxidative stress resistance in Nile tilapia. Our findings highlight the potential of CH as a feed additive to improve Nile tilapia fingerlings’ health and productivity in sustainable biofloc aquaculture.

## Figures and Tables

**Table 1:** Growth performances and feed efficiency in Nile tilapia (*Oreochromis niloticus*) fingerlings fed diets with increasing chitosan (CH) levels for 4 and 8 weeks. Data is shown as mean ± SEM. Different letters (a–b) indicate significant differences between dietary groups. Diets: CH0, 0 mL·kg^−1^ COS (control); CH5, 5 mL·kg^−1^; CH10, 10 mL·kg^−1^; CH20, 20 mL·kg^−1^; CH40, 40 mL·kg^−1^ of chitosan.

	CH0	CH5	CH10	CH20	CH40	*p*-Value
**IW (g)**	13.48 ± 0.03	13.66 ± 0.05	13.55 ± 0.03	13.56 ± 0.08	13.47 ± 0.05	0.499
**FW (g)**						
4 weeks	29.45 ± 1.33	28.97 ± 0.98	31.72 ± 0.28	29.06 ± 0.38	28.87 ± 1.27	0.470
8 weeks	50.44 ± 0.67 ^b^	50.23 ± 0.62 ^b^	50.69 ± 0.54 ^a^	50.60 ± 1.20 ^b^	50.29 ± 1.95 ^b^	0.049
**WG (g)**						
4 weeks	15.97 ± 1.26	15.30 ± 0.93	18.17 ± 0.44	15.49 ± 0.45	15.40 ± 1.20	0.228
8 weeks	36.96 ± 1.30 ^b^	36.56 ± 0.04 ^b^	40.14 ± 0.83 ^a^	37.03 ± 0.85 ^b^	36.81 ± 0.47 ^b^	0.050
**SGR (%/day)**						
4 weeks	2.60 ± 0.13 ^ab^	2.50 ± 0.10 ^b^	2.83 ± 0.07 ^a^	2.54 ± 0.06 ^ab^	2.54 ± 0.13 ^ab^	0.032
8 weeks	2.20 ± 0.04 ^b^	2.17 ± 0.00 ^b^	2.29 ± 0.03 ^a^	2.19 ± 0.04 ^b^	2.19 ± 0.01 ^b^	0.047
**FCR**						
4 weeks	0.76 ± 0.03 ^a^	0.76 ± 0.01 ^a^	0.75 ± 0.03 ^a^	0.75 ± 0.02 ^a^	0.75 ± 0.02 ^a^	0.479
8 weeks	1.02 ± 0.01 ^a^	1.07 ± 0.04 ^a^	1.08 ± 0.04 ^a^	1.02 ± 0.03 ^a^	1.07 ± 0.08 ^a^	0.855
**SR (%)**						
4 weeks	96.67 ± 1.67 ^a^	95.00 ± 2.89 ^a^	98.33 ± 3.33 ^a^	98.33 ± 1.67 ^a^	98.33 ± 1.67 ^a^	0.046
8 weeks	96.67 ± 1.67 ^a^	95.00 ± 2.89 ^a^	98.33 ± 3.33 ^a^	98.33 ± 1.67 ^a^	98.33 ± 1.67 ^a^	0.046

IW (g) = initial weight; FW (g) = final weight; WG (g) = weight gain; SGR (%) = specific growth rate; FCR = feed conversion ratio; SR (%) = survival rate.

**Table 2:** Skin mucus lysozyme and peroxidase activities in Nile tilapia (*Oreochromis niloticus*) fingerlings fed diets with increasing chitosan (CH) levels for 4 and 8 weeks. Data is shown as mean ± SEM. Different letters (a–b) indicate significant differences between dietary groups. Diets: CH0, 0 mL·kg^−1^ (control); CH5, 5 mL·kg^−1^; CH10, 10 mL·kg^−1^; CH20, 20 mL·kg^−1^; CH40, 40 mL·kg^−1^ of chitosan.

		CH0	CH5	CH10	CH20	CH40	*p*-Value
4 weeks	SMLA	0.217 ^a^ ± 0.01	0.205 ^b^ ± 0.01	0.211 ^ab^ ± 0.01	0.195 ^b^ ± 0.01	0.201 ^b^ ± 0.01	0.048
	SMPA	0.313 ^a^ ± 0.02	0.287 ^b^ ± 0.01	0.304 ^ab^ ± 0.01	0.261 ^b^ ± 0.02	247 ^b^ ± 0.03	0.050
8 weeks	SMLA	0.249 ^a^ ± 0.01	0.215 ^b^ ± 0.01	0.220 ^ab^ ± 0.01	0.201 ^b^ ± 0.01	0.215 ^b^ ± 0.01	0.001
	SMPA	0.213 ^a^ ± 0.04	0.193 ^b^ ± 0.06	0.204 ^ab^ ± 0.06	0.181 ^b^ ± 0.02	147 ^b^ ± 0.02	0.036

SMLA (µg mL^−1^) = skin mucus lysozyme activity; SMPA (µg mL^−1^) = skin mucus peroxidase activity.

**Table 3:** Serum lysozyme and peroxidase activities in Nile tilapia (*Oreochromis niloticus*) fingerlings fed diets with increasing chitosan (CH) levels for 4 and 8 weeks. Data is shown as mean ± SEM. Different letters (a–c) indicate significant differences between dietary groups by one-way ANOVA and Duncan’s test (*p* < 0.05). Diets: CH0, 0 mL·kg^−1^ COS (control); CH5, 5 mL·kg^−1^; CH10, 10 mL·kg^−1^; CH20, 20 mL·kg^−1^; CH40, 40 mL·kg^−1^ of chitosan.

		CH0	CH5	CH10	CH20	CH40	*p*-Value
4 weeks	SL	0.297 ^a^ ± 0.01	0.275 ^bc^ ± 0.01	0.289 ^ab^ ± 0.01	0.258 ^c^ ± 0.01	0.286 ^b^ ± 0.01	0.038
	SP	0.443 ^b^ ± 0.02	0.449 ^b^ ± 0.02	0.501 ^a^ ± 0.01	0.388 ^c^ ± 0.01	0.392 ^c^ ± 0.01	0.015
8 weeks	SL	0.248 ^a^ ± 0.01	0.225 ^bc^ ± 0.01	0.240 ^ab^ ± 0.01	0.212 ^c^ ± 0.01	0.231 ^b^ ± 0.01	0.049
	SP	0.477 ^b^ ± 0.05	0.438 ^abc^ ± 0.04	0.522 ^a^ ± 0.03	0.377 ^c^ ± 0.02	0.392 ^bc^ ± 0.03	0.041

SL: serum lysozyme activity (μg mL^−1^); SP: serum peroxidase activity (μg mL^−1^).

**Table 4:** The formulation and proximate composition of the experimental diets (g/kg of the basal diets).

	CH0	CH5	CH10	CH20	CH40
Fish meal	200	200	200	200	200
Corn meal	150	150	150	150	150
Soybean meal	390	390	390	390	390
Wheat flour	70	70	70	70	70
Rice bran	150	150	150	150	150
Soybean oil	2	2	2	2	2
Chitosan solution (mL)	0	5	10	20	40
Binder	20	20	20	20	20
Premix ^1^	10	10	10	10	10
Vitamin C 98%	8	8	8	8	8
Total (g)	1000	1000	1000	1000	1000
Proximate composition of the experimental diets (%)					
Crude protein	32.80	32.00	32.60	32.40	32.50
Crude lipid	2.85	2.75	2.63	2.78	2.88
Fiber	3.68	3.74	3.44	3.72	3.55
Ash	7.59	7.86	7.75	7.35	7.91
Dry matter	99.16	98.40	98.35	97.77	97.54
Gross Energy (cal/g)	4273.00	4261.50	4253.90	4262.00	4245.00

^1^ Vitamin and trace mineral mix supplemented as follows (IU kg^−1^ or g kg^−1^ diet): retinyl acetate 1,085,000 IU; cholecalciferol 217,000 IU; D, L-a-tocopherol acetate 0.5 g; thiamin nitrate 0.5 g; pyridoxine hydrochloride 0.5 g; niacin 3 g; folic 0.05 g; cyanocobalamin 10 g; Ca pantothenate 1 g kg^−1^; inositol 0.5 g; zinc 1 g; copper 0.25 g; manganese 1.32 g; iodine 0.05 g; sodium 7.85 g.

**Table 5:** Primer sequences used for quantitative real-time PCR.

Genes	Primer Sequence (5′-3′)	Tm (°C)	Product Size (bp)	Reference
*18S-rRNA*	GTGCATGGCCGTTCTTAGTT CTCAATCTCGTGTGGCTGAA	60	150	XR_003216134
*IL-1*	GTCTGTCAAGGATAAGCGCTG ACTCTGGAGCTGGATGTTGA	59	200	XM_019365844
*IL-8*	CTGTGAAGGCATGGGTGTG GATCACTTTCTTCACCCAGGG	59	196	NM_001279704
*LBP*	ACCAGAAACTGCGAGAAGGA GATTGGTGGTCGGAGGTTTG	59	200	XM_013271147
*GST-α*	ACTGCACACTCATGGGAACA TTAAAAGCCAGCGGATTGAC	60	190	NM_001279635
*GPX*	GGTGGATGTGAATGGAAAGG CTTGTAAGGTTCCCCGTCAG	60	190	NM_001279711
*GSR*	CTGCACCAAAGAACTGCAAA CCAGAGAAGGCAGTCCACTC	60	172	XM_005467348

## Data Availability

The data presented in this study are available on request from the corresponding author.
